# Exposure to Hyperbaric Oxygen Intensified Vancomycin-Induced Nephrotoxicity in Rats

**DOI:** 10.1371/journal.pone.0152554

**Published:** 2016-04-19

**Authors:** Itay M. Sabler, Matitiahu Berkovitch, Judith Sandbank, Eran Kozer, Zahi Dagan, Michael Goldman, Hilla Bahat, Kobi Stav, Amnon Zisman, Baruch Klin, Ibrahim Abu-Kishk

**Affiliations:** 1 Department of Urology, Assaf Harofeh Medical Center, Zerifin, affiliated to the Sackler School of Medicine, Tel-Aviv University, Tel-Aviv, Israel; 2 Clinical Pharmacology Unit, Assaf Harofeh Medical Center, Zerifin, affiliated to the Sackler School of Medicine, Tel-Aviv University, Tel-Aviv, Israel; 3 Pathology Department, Assaf Harofeh Medical Center, Zerifin, affiliated to the Sackler School of Medicine, Tel-Aviv University, Tel-Aviv, Israel; 4 Pediatric Division, Assaf Harofeh Medical Center, Zerifin, affiliated to the Sackler School of Medicine, Tel-Aviv University, Tel-Aviv, Israel; 5 Pediatric Nephrology Service, Assaf Harofeh Medical Center, Zerifin, affiliated to the Sackler School of Medicine, Tel-Aviv University, Tel-Aviv, Israel; Emory University, UNITED STATES

## Abstract

It has been suggested that oxidative stress is a potential mechanism for vancomycin-induced nephrotoxicity and hyperbaric oxygen therapy (HBO) has been shown to be effective in treating renal toxicity that has been pharmacologically induced in animal models. The aim of this study was to investigate the effect of HBO therapy on vancomycin-induced nephrotoxicity in rats. The study group comprised 36 Sprague Dawley male rats. We treated 30 with 500 mg/kg of intraperitoneal vancomycin once a day for 7 days. Half of these rats received a daily 1-hour treatment with HBO at 2 Atmospheres (ATM) on the same 7 days and formed the HBO+ group. The other 15 subjects received no HBO treatment (HBO- group). The remaining six rats served as the control group, three received HBO treatments alone and no treatment was administered to the other three rats. Laboratory results were obtained on day 8 and the intervention and control groups were compared. Rats in the HBO+ group gained less weight than the HBO- group (11.6 grams vs 22.6 grams; P = 0,008) and had significantly higher serum blood urea nitrogen (99.6 vs 52.6 mg/dL; P<0.001), serum creatinine (0.42 vs 0.16 mg/dL; P = 0.001) and magnesium (3.6 vs 3.1mg/dL; P = 0.014). The vancomycin blood levels were also higher in the HBO+ group (27.8 vs 6.7 μg/mL; P = 0.078). There were no pathological kidney changes in the control group. All the kidneys from the treated groups (vancomycin +HBO and vancomycin HBO-) showed moderate to severe histopathological changes with no statistical significance between them. This study demonstrated that exposure to hyperbaric oxygen intensified vancomycin-induced nephrotoxicity in rats.

## Introduction

Vancomycin is a glycopeptide antibiotic that has been used to treat *methicillin resistant S*. *Aureus* infections for more than 50 years. The reported incidence of vancomycin-induced nephrotoxicity is 5–25% [1]. One study quantified the nephrotoxic vancomycin dose by measuring urinary cell and enzyme excretion in rats. It found that an intravenous injection of 25 mg/kg/day induced increased renal cell elimination and intraperitoneal vancomycin of 100 mg/kg/day induced the elimination of tubule cells, elevated serum creatinine levels and resulted in widespread tubular necrosis [2].

The exact mechanism of vancomycin-induced nephrotoxicity is unknown, but it has been suggested that oxidative stress and hypoxia may be involved in the pathogenesis [3,4]. Since vancomycin is secreted by the renal proximal tubule, it is hypothesized that this is the site where vancomycin-induced nephrotoxicity takes place [5].

Some studies have reported that exposure to 100% oxygen at two to three times the atmospheric pressure at sea level results in increased arterial oxygen tension and that it has a number of beneficial biochemical, cellular, and physiologic effects [6,7]. It has also been postulated in other studies that hyperbaric oxygen therapy (HBO) can induce protection against oxidative insults [8,9].

Since HBO may reverse the effects of vancomycin-induced renal toxicity by improving the effects of hypoxia and oxidative stress, we aimed to assess the effect of HBO treatment on vancomycin-induced renal failure in a rat model.

## Materials and Methods

The study was approved by the local animal care committee (Assaf Harofeh Medical Center Animal Care Committee: Permit Number: 01/2013). The rats were handled in strict adherence to the Institutional Animal Care and Use Committee (IACUC) standards.

All procedures were performed under sedation with carbon dioxide (CO2), and all efforts were made to minimize suffering. Each animal was placed in an empty clean chamber; the flow of CO2 from the gas cylinder was started at a rate that would displace 10–30% of the chamber volume per minute. This calculated rate would allow a slow increase in the concentration of CO2 to develop, but would not cause noise or be perceived as a harsh “wind” to the animals. As gas levels rose to 40–50%, unconsciousness occurred as indicated by a loss of the righting reflex. At this point, the procedures with the rats were performed, for example intraperitoneal injection of medication or withdrawal of blood by cardiac puncture. At the end of the experiment protocol, the animals were sacrificed by cervical dislocation after CO2 sedation.

The rats were monitored twice daily by a staff member authorized by the institutional veterinarian. If one of the following conditions occurred during the experiment, the animal was sacrificed and experiment termination considered, according to the decision of the institutional veterinarian: signs of suffering or pain that could not be treated with analgesics; significant changes in physiological parameters (breathing, heart rate, social behavior); other signs of distress (apathy, prolonged lying, aggressive behavior, self harm, anorexia, hyperactivity); refusal to eat or drink independently for 48h and 24h respectively; an infection, swelling or inflammation that could not be treated; loss of more than 10% of baseline weight.

### Treatment of Animals

We studied 36 male Sprague-Dawley rats weighing 170–180 grams that were double-housed in shoe-box type cages with free access to food and water. Vancomycin (Edicin, Sandoz İlaç, Istanbul, Turkey) was diluted with 20 ml of sterile water, according to the manufacturer's instructions, and the final concentration for the injections was 50 mg/ml. The rats were weighed on digital scales and the total dose of 500 mg/kg/day, given to each rat as a single daily injection, was individually calculated. The solution was delivered as an intraperitoneal injection under sedation with CO2.

HBO was administered using a locally manufactured hyperbaric chamber designed to contain 20 rats. HBO exposure consisted of administering 100% oxygen at 2 Atmospheres (ATM) for 70 minutes, including 5 minutes for compression and 5 minutes for decompression.

### Serum Creatinine, Urea and Magnesium Measurement

We measured any elevations in the serum creatinine, urea and magnesium levels using the Roche/Hitachi modular P800 autoanalyser (Roche Diagnostics, Mannheim, Germany) and elevated potassium levels using the Roche/Hitachi modular ISE 900 autoanalyser (Roche Diagnostics, Mannheim, Germany). Acute renal failure was defined as doubling of creatinine and urea plasma levels [10,11].

### The Study Protocol

The study protocol was divided into two phases. The first phase was designed to determine the vancomycin dose required to induce renal failure, which was defined as doubling of plasma creatinine levels in comparison to the control group [10,11]. During the second phase, we induced nephrotoxicity by using the vancomycin dose identified during the first phase and exposed the rats to HBO.

Thirty of the rats were then randomly divided into two groups of 15. For the first seven consecutive days of the study, all 30 rats received one intraperitoneal injection per day using the nephrotoxic vancomycin dose. Fifteen of these rats (HBO+) were also subjected to a daily session of HBO exposure starting on day one, while the other 15 (HBO-) were not. The remaining six rats served as the control group, three rats received HBO treatments alone and no treatment was administered to the remaining three. On day 8, the rats were sedated with CO2 and blood was drawn by cardiac puncture. Blood urea nitrogen, serum creatinine, electrolytes and trough plasma vancomycin levels were measured and all the animals were sacrificed by cervical dislocation. The rat kidneys were immediately harvested for histologic examination.

### Histopathologic Examination

The renal samples were prepared for light microscopy, halved through the hilum, along the long axis perpendicular to the capsule, and fixed in buffered 10% formalin, embedded in paraffin and stained with Hematoxylin and eosin stain.

The study was pathologist-blinded and the histopathologic examination was performed by an experienced pathologist.

The entire cortical areas of all the specimens were evaluated in order to assess the extent of the damage induced by the different treatment protocols.

The following features were monitored: proximal tubular cell degeneration (eosinophilia) and regeneration (basophilia), tubular dilatation, intra tubular casts and interstitial inflammation.

The changes were monitored using a six-tier grading system, as follows: Grade 0 –normal, grade 1 –minimal changes only (weak and focal), grade 2 - <25% of the cortex underwent histopathological changes, grade 3–25–50%, grade 4–50–75%, and grade - >75% [12].

### Statistical analysis

Descriptive statistics were used to describe the various groups. Continuous variables were first tested for normality. Groups were compared using the student t-test or Mann Whitney test for continuous variables and Fisher’s exact test for discrete variables. The level of significance for all tests was a p value of 0.05. Statistical analysis was conducted using the 21^st^ edition of the SPSS computer program (IBM Corp Armonk, NY).

## Results

### Body weight and survival

All the rats in the control group survived and gained weight from 180 ± 6.3 gram on day 1 to 218 ± 7 gram on day 8. One rat in the HBO- control group died immediately after injection of vancomycin on the fourth day of the experiment. The mean animal body weight on day 1 was 179.3 ± 5.9 grams for the HBO group and 180 ± 7.6 grams for the HBO- group. At day 8, the mean weight was 190.9 ± 12.6 grams for the HBO+ group and 202.6 ± 10.6 gr for the HBO- group. The HBO+ group gained significantly less weight during the study period than the HBO- group (11.6 ± 6.8 grams vs 22.6 ± 11.6 grams; P = 0.008) (Table 1).

**Table 1:** Comparison of weight, renal function, electrolytes and trough vancomycin levels between the treatment arms.

	HBO-		SD	HBO+		SD	P Value
	Median	Mean		Median	Mean		
Weight day 1	180	180	7.4	181	179.39	5.9	
Weight day 8	205	202.6	10.6	189	190.9	12.6	0.008
K (mg/dL)	5.8	5.8	0.4	5.8	5.8	0.3	0.483
Na (mg/dL)	142	141.9	1.8	142	141.4	1.2	0.485
Ca (mg/dL)	10.4	10.2	0.9	10.7	10.6	0.5	0.044
Mg (mg/dL)	2.9	3.1	0.5	3.6	3.6	0.6	0.014
Urea (mg/dL)	48.9	52.6	15.4	82	99.6	48.1	<0.001
Creatinin (mg/dL)	0.17	0.16	0.06	0.32	0.42	0.38	0.001
VMC (μg/mL)	5	6.7	4.9	18.6	27.8	30.2	0.078

K = potassium, Na = sodium, Ca = calcium, Mg = magnesium, SD = standard deviation

### Biochemical parameters

Biochemical values of the control group were as follows: urea (mg/dl) 19–21, creatinine (mg/dl) 0.08–0.1,calcium (mg/dl) 10.7–11.1,sodium (mmol/L) 137–141,potassium (mmol/L) 4.1–5.2. Rats exposed to HBO had significantly higher serum blood urea nitrogen levels (99.6 ± 48.1 vs 52.6 ± 15.4 mg/dL; P<0.001) and serum creatinine levels (0.42 ± 0.38 vs 0.16 ± 0.06 mg/dL; P = 0.001). Plasma magnesium levels were 3.6 ± 0.6 mg/dL vs 3.1 ± 0.5 mg/dL, (P = 0.014). The mean trough plasma vancomycin level was 27.8 ± 30.2 μg/mL in the HBO+ group and 6.7 ± 4.9 μg/mL in the HBO- group (P = 0.078).

No statistically significant differences were found between the two groups in terms of plasma sodium, calcium or potassium levels (Table 1).

### Histologic evaluation

We measured the kidney changes using the six-tiered scale described above. Table 2 shows the distribution of pathological grading between the various groups.

**Table 2:** The distribution of pathological grading between the groups.

Grade	Control No treatment	Control HBO	Vancomycin	Vancomycin +HBO
0	3	3	0	0
1	0	0	0	2
2	0	0	5	5
3	0	0	8	4
4	0	0	1	1
5	0	0	0	3

**Fig 1:**
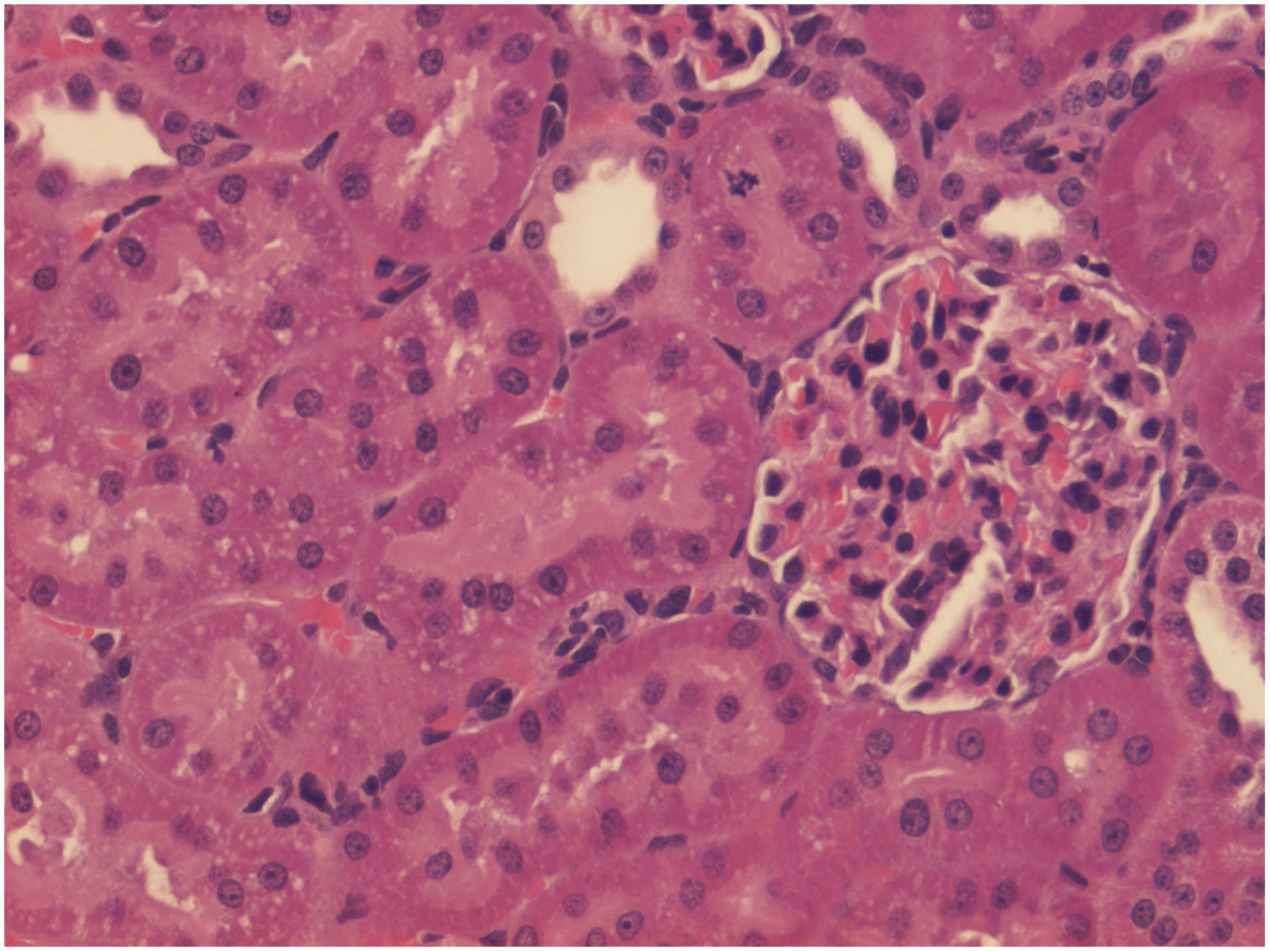
Histologic evaluation. Normal kidney, appropriate for control groups (No treatment group and HBO alone group). *HE = hematoxylin and eosin*.

**Fig 2:**
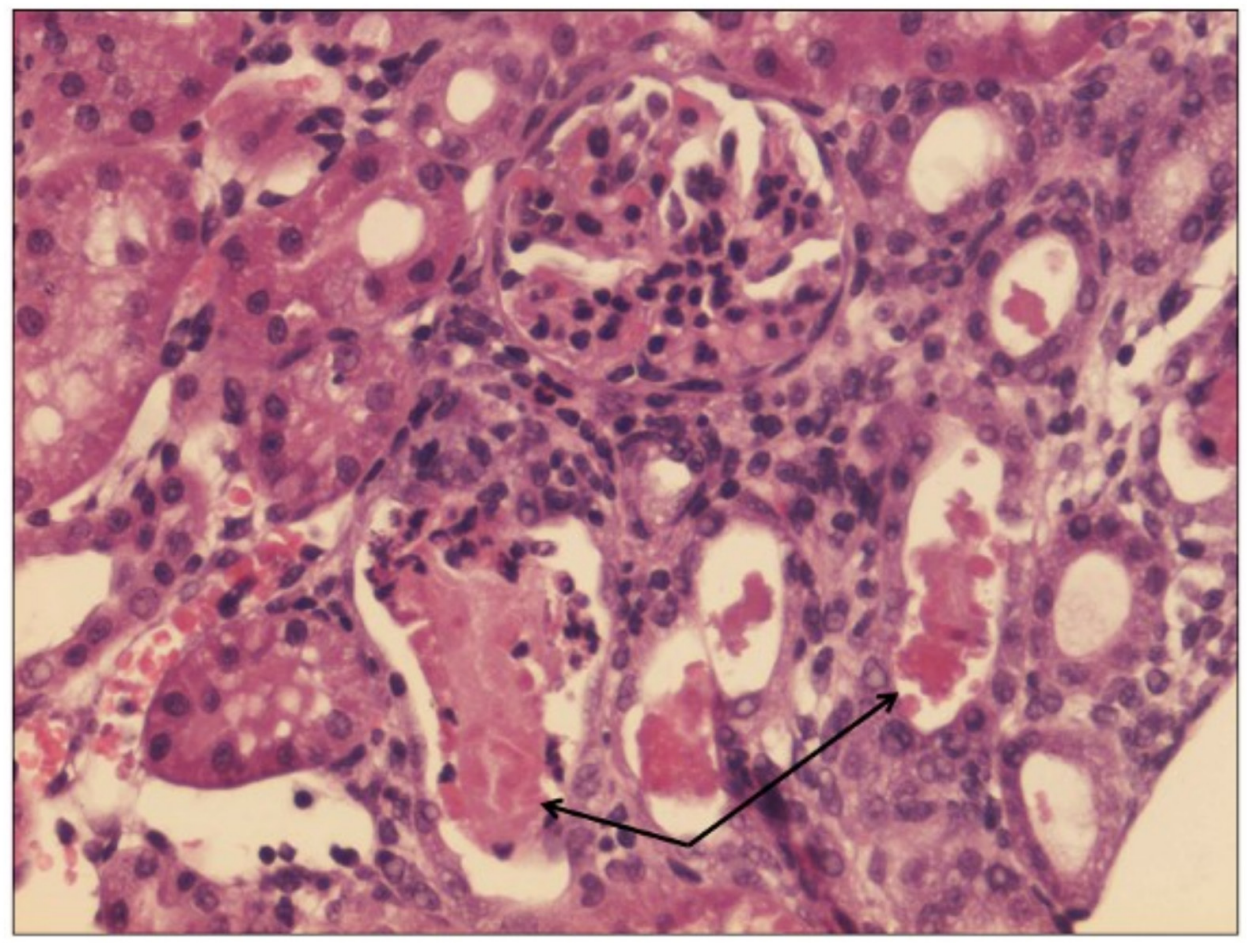
Histologic evaluation. Vancomycin only (HE x20) PMN leukocytes in a few cortical tubules (arrows). *HE = hematoxylin and eosin*, *PMN = polymorphonuclear*.

**Fig 3:**
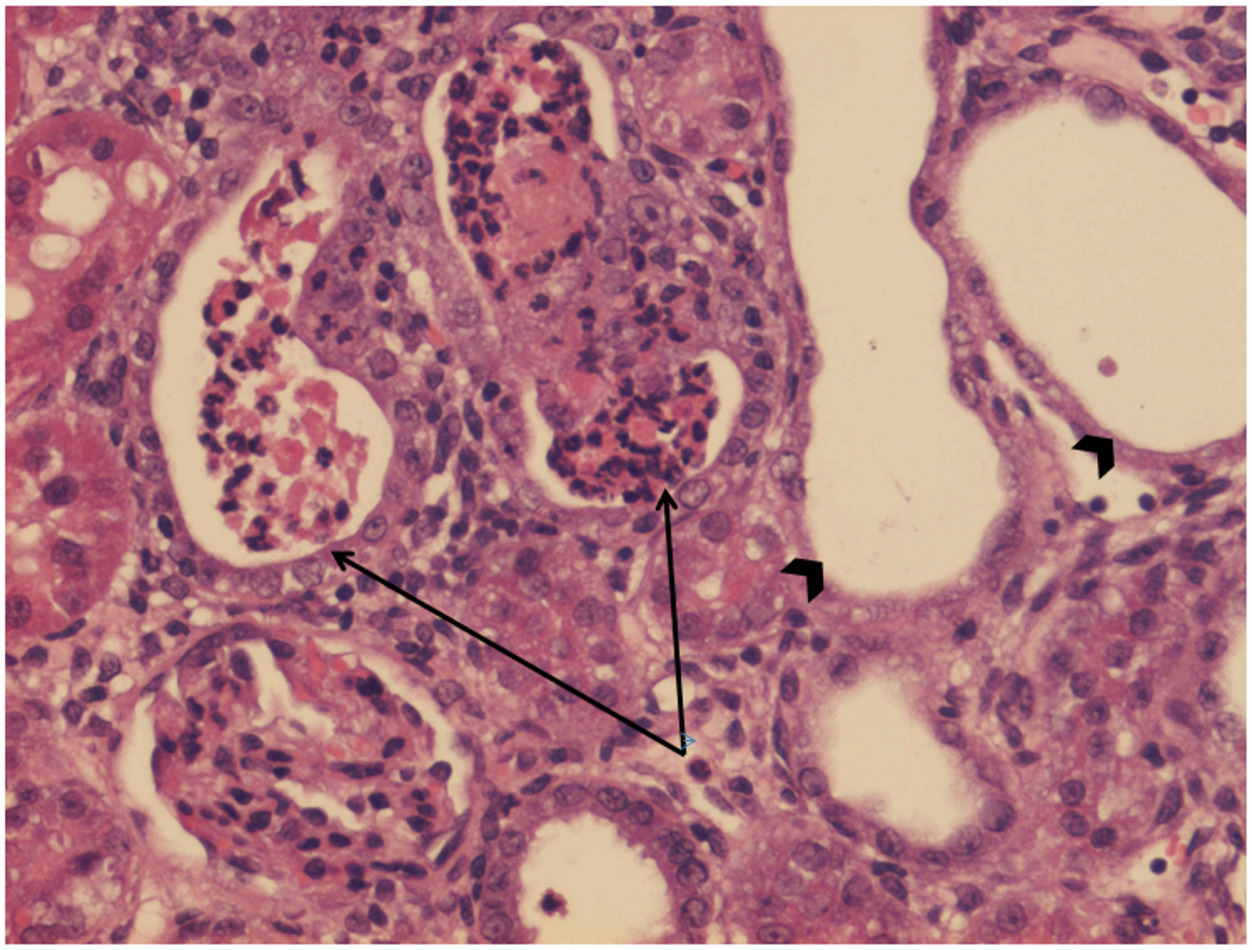
Histologic evaluation. Vancomycin and hyperbaric oxygen (HE x20) PMN leukocytes (arrows), dilatation of numerous cortical tubules (arrow heads). *HE = hematoxylin and eosin*, *PMN = polymorphonuclear*.

No treatment group and HBO alone group had normal kidneys (Fig 1) and no significant differences were found between them. There were significant differences between the control groups and the groups treated with vancomycin and vancomycin + HBO (P = 0.003 and P = 0.002 respectively). Figs 2 and 3 illustrate the pathological changes in the groups treated with vancomycin and vancomycin + HBO. There were no significant differences between the groups treated with vancomycin alone and vancomycin + HBO (P = 0.98).

## Discussion

The findings of the present study provide novel information on the further deterioration in renal function in rats exposed to HBO. Our data clearly demonstrate that HBO nephroprotection does not take place in vancomycin-induced nephrotoxicity.

Elimination of vancomycin is almost exclusively renal, as it is excreted in the glomeruli and, to some extent, via active tubular secretion [5]. The significantly elevated magnesium levels found in the serum of animals who received HBO may support the theory that the proximal tubules are the primary site of nephrotoxicity in vancomycin-treated rats.

Although vancomycin levels tended to be higher in the HBO+ group in our study, the difference was not statistically significant. Vancomycin requires energy dependent transport from the blood to the tubular cells across the basolateral membrane [13]. Some studies have suggested that oxidative stress might underline the pathogenesis of vancomycin-induced toxicity [3,4]. Gene expression analyses have suggested involvement of oxidative stress and mitochondrial damage in vancomycin-induced kidney injury as a potential contribution of complement pathway and inflammation in vancomycin-induced renal toxicity. Severe vancomycin renal toxicity may present histologically as tubulo-interstitial nephritis, sometimes with granulomas [13,14]. In other words, hypoxia that occurs as a result of the above-mentioned processes probably plays the major role in vancomycin-induced renal function deterioration. This suggested mode of action was the reason why we decided to test whether HBO had a corrective action after vancomycin renal injury.

HBO therapy has been used to treat a number of medical conditions [7]. According to its proposed mechanism of action, HBO produces a hyperoxic state and increases oxygenation in the tissues with upregulated oxygen demand. Using the example of rat tissues, HBO has been shown to inhibit neutrophil adherence to the wall of ischemic vessels and to decrease post-ischemic vasoconstriction in skin grafts [15]. Furthermore, it has been shown that HBO had beneficial anti-inflammatory effects on experimental colitis in rats [16]. In several experimental studies, HBO has been claimed to exert beneficial effects by partly increasing the activity of Cu/Zn superoxide dismutase (Cu/Zn-SOD) and other antioxidant cellular defense mechanisms, thereby altering the balance between the generation and removal of oxygen-free radicals [17,18].

HBO is capable of inducing cellular protection against ischemia. These cell protective mechanisms are still poorly understood, but studies have shown that they include the expression of stress-inducible proteins, including the anti-apoptotic protein Bcl-2 and the free radical scavenger Mn-super oxide dismutase [19]. Our previous animal experiment showed no change in renal function in rats that were only exposed to HBO [20]. Despite the fact that different studies have found HBO to be nephroprotective, S Aydinoz et al found incremental deterioration of renal function in rats treated with both HBO and cisplatin [21].

When oxygen is breathed at high partial pressures, such as in HBO exposure, a hyperoxic condition rapidly evolves within the most vascularised tissues. During times of environmental stress, levels of reactive oxygen species can increase dramatically, producing oxidative stress and leading to cell structure damage. High concentrations of oxygen can also increase the formation of other free radicals, such as nitric oxide, peroxynitrite and trioxidane, which harm DNA and other biomolecules [22–24]. Although there are many antioxidant systems, such as glutathione, that guard against oxidative stress, these systems are eventually overwhelmed, leading to cell damage and cell death [25–27]. The mechanism mentioned above may be responsible for the renal damage observed in this study.

The exact mechanism of vancomycin-induced nephrotoxicity has not been fully identified. One study suggested that vancomycin exposure induces a proliferative cell response as evidenced by an increased cell number and increased total protein [28]. In their study, pretreatment with a mitogen-activated protein kinase inhibitor inhibited these effects. This effect of vancomycin was accompanied by increased oxygen consumption and enhanced mitochondrial respiration [28]. It is possible that HBO aggravates this process by increasing the dissolving oxygen in tissues.

Another study that aimed to elucidate the mechanism of vancomycin transport in the kidney revealed that vancomycin nephrotoxicity may be due to entry through the basolateral membrane and the absence of mediated egress at the brush border membrane [29]. This study demonstrated that vancomycin is a substrate for the renal basolateral organic cation transport system and can be concentrated in the cells via this mechanism. Once inside, vancomycin can cause nephrotoxicity due to the absence of a mediated transport mechanism across the brush border membrane. The absence of such a mechanism could explain why HBO did not help in the present study.

Pressure that is higher than barometric pressure, like in pneumoperitoneum, has been shown to cause transient renal function deterioration in animals [30]. Increased pressure may cause compression of renal vessels or parenchyma and further deterioration in drug induced renal damage. The significant elevation of trough vancomycin levels noted in the HBO+ group is interesting, as it points to fact that the animal kidneys were exposed to increased amounts of vancomycin and that this may have led to even more pronounced acute renal failure.

Further research is needed to improve our understanding of the mechanism by which HBO interacts with different drugs. Vancomycin is an important antimicrobial agent that is widely used in clinical practice for treating Methicillin resistant S.Aureus infections. Nephrotoxicity is a typical side effect of the drug and the drug dose should be altered or stopped if this happens. As a result of our study, we are urging clinicians to be cautious about providing both vancomycin and HBO to patients at the same time until more precise clinical data is available that clarifies whether the findings of our animal study would also apply to human subjects.

There are some limitations to our study, as we examined indirect markers of glomerular filtration rate, namely serum creatinine, blood urea nitrogen and blood electrolytes. These chemical markers are easy to obtain, cheap and readily available, but the measurement of plasma creatinine is not the optimal method and more sensitive markers, such as beta 2 microglobulin and cystatin c, do exist. Oxidative markers, that would further clarify the findings, were not obtained. Our study also lacked two more experimental arms that could add to our understanding of why renal failure occurred in rats exposed to vancomycin and HBO: pure oxygen at 1 ATM and fresh air at 2 ATM.

## Conclusions

In this rat model study, HBO therapy intensified the acute renal injury inflicted by vancomycin, as reflected by serum creatinine and urea measurements and histopathological findings. We found elevated, although not statistically significant, vancomycin trough levels in the animals treated with HBO compared to the animals who did not receive HBO. However, further research is needed to establish whether human patients would react in the same way. Further research is also needed into the mechanism by which a combination of HBO and vancomycin causes renal damage, as this is not completely understood.

## Supporting Information

S1 Results Comparison of body weight follow up during the experiment, renal function, electrolytes and trough vancomycin levels between the treatment arms (with and without HBO). (XLS)

S2 Results Control Group. Body weight follow up during the experiment, renal function, electrolytes in the control group (no treatment and HBO alone treatment).
(XLS)

S3 Results Pathological Grading. Comparison of pathological score between the all groups.
(XLS)

S1 Table Comparison of weight, renal function, electrolytes and trough vancomycin levels between the treatment arms (with and without HBO). (XLS)

## References

[1] Levine DP. Vancomycin: a history. Clin Infect Dis. 2006; 42(Suppl 1): S5–S12. 16323120 10.1086/491709

[2] Marre R, Schulz E, Anders T, Sack K. Renal tolerance and pharmacokinetics of vancomycin in rats. J Antimicrob Chemother. 1984; 14: 253–260. 6490570 10.1093/jac/14.3.253

[3] Nishino Y, Takemura S, Minamiyama Y, Hirohashi K, Ogino T, Inoue M, et al Targeting superoxide dismutase to renal proximal tubule cells attenuates vancomycin-induced nephrotoxicity in rats. Free Radic Res. 2003; 37: 373–379. 12747731 10.1080/1071576031000061002

[4] Oktem F, Arslan MK, Ozguner F, Candir O, Yilmaz HR, Ciris M, et al In vivo evidences suggesting the role of oxidative stress in pathogenesis of vancomycin-induced nephrotoxicity: protection by erdosteine. Toxicology. 2005; 215: 227–233. 16112787 10.1016/j.tox.2005.07.009

[5] Appel GB, Given DB, Levine LR, Cooper GL. Vancomycin and the kidney. Am J Kidney Dis. 1986; 8: 75–78. 3526874 10.1016/s0272-6386(86)80116-0

[6] Tibbles PM, Edelsberg JS. Hyperbaric-oxygen therapy. N Engl J Med. 1996; 334: 1642–1648. 8628361 10.1056/NEJM199606203342506

[7] Gill AL, Bell CN. Hyperbaric oxygen: its uses, mechanisms of action and outcomes. QJM. 2004; 97: 385–395. 15208426 10.1093/qjmed/hch074

[8] Chen LF, Tian YF, Lin CH, Huang LY, Niu KC, Lin MT. Repetitive hyperbaric oxygen therapy provides better effects on brain inflammation and oxidative damage in rats with focal cerebral ischemia. J Formos Med Assoc. 2014; 113: 620–628. 10.1016/j.jfma.2014.03.012 24787662

[9] Lin H, Chang CP, Lin HJ, Lin MT, Tsai CC. Attenuating brain edema, hippocampal oxidative stress, and cognitive dysfunction in rats using hyperbaric oxygen preconditioning during simulated high-altitude exposure. J Trauma Acute Care Surg. 2012;72: 1220–1227. 10.1097/TA.0b013e318246ee70 22673248

[10] Huang SH, Macnab JJ, Sontrop JM, Filler G, Gallo K, Lindsay RM, et al Performance of the creatinine-based and the cystatin C-based glomerular filtration rate (GFR) estimating equations in heterogenous sample of patients referred for nuclear GFR testing. Transl Res. 2011; 157: 357–367. 10.1016/j.trsl.2011.01.002 21575920

[11] Kellum JA, Levin N, Bouman C, Lameire N. Developing a consensus classification system for acute renal failure. Curr Opin Crit Care. 2002; 8: 509–514. 12454534 10.1097/00075198-200212000-00005

[12] Aronoff GR, Sloan RS, Dinwiddie CB Jr, Glant MD, Fineberg NS, LKuft FC. Effects of vancomycin on renal function in rats. Antimicrob Agent Chemother. 1981; 19: 306–308. 10.1128/aac.19.2.306 PMC181415 7347562

[13] Nakamura T, Takano M, Yasuhara M, Inui K. In-vivo clearance study of vancomycin in rats. J Pharm Pharmacol. 1996; 48: 1197–2003. 8961172 10.1111/j.2042-7158.1996.tb03920.x

[14] Dieterich C, Puey A, Lin S, Swezey R, Furimsky A, Fairchild D, et al Gene expression analysis reveals new possible mechanisms of vancomycin-induced nephrotoxicity and identifies gene markers candidates. Toxicol Sci. 2009; 107: 258–269. 10.1093/toxsci/kfn203 18930951 PMC2638642

[15] Hong S, Valderrama E, Mattana J, Shah HH, Wagner JD, Esposito M, et al Vancomycin-induced acute granulomatous interstitial nephritis: therapeutic options. Am J Med Sci. 2007; 334: 296–300. 18030187 10.1097/MAJ.0b013e3180a6ec1e

[16] Zamboni WA, Roth AC, Russell RC, Graham B, Suchy H, Kucan JO. Morphologic analysis of the microcirculation during reperfusion of ischemic skeletal muscle and the effect of hyperbaric oxygen. Plast Reconstr Surg.1993; 91: 1110–1123. 8479978 10.1097/00006534-199305000-00022

[17] Rachmilewitz D, Karmeli F, Okon E, Rubenstein I, Better OS. Hyperbaric oxygen: a novel modality to ameliorate experimental colitis. Gut.1998; 43: 512–518. 9824579 10.1136/gut.43.4.512 PMC1727276

[18] Gregorevic P, Lynch GS, Williams DA. Hyperbaric oxygen modulates antioxidant enzyme activity in rat skeletal muscles. Eur J Appl Physiol. 2001; 86: 24–27. 11820317 10.1007/s004210100503

[19] Mrsić-Pelcić J, Pelcić G, Vitezić D, Antoncić I, Filipović T, Simonić A et al Hyperbaric oxygen treatment: the influence on the hippocampal superoxide dismutase and Na+,K+-ATPase activities in global cerebral ischemia-exposed rats. Neurochem Int. 2004; 44: 585–594. 15016473 10.1016/j.neuint.2003.10.004

[20] Berkovitch M, Tsadik R, Kozer E, Abu-Kishk I. The effect of hyperbaric oxygen therapy on kidneys in rat model. Scientific World Journal. 2014; Open access, available. 10.1155/2014/105069 PMC4142719 25177712

[21] Aydinoz S, Uzun G, Cermik H, Atasoyu EM, Yildiz S, Karagoz B, et al, Effects of different doses of hyperbaric oxygen on cisplatin-induced nephrotoxicity. Ren Fail. 2007; 29: 257–263. 17497437 10.1080/08860220601166487

[22] Wada K, Miyazawa T, Nomura N, Yano A, Tsuzuki N, Nawashiro H, et al Mn-SOD and Bcl-2 expression after repeated hyperbaric oxygenation. Acta Neurochir Suppl. 2000; 76: 285–290. 11450026 10.1007/978-3-7091-6346-7_59

[23] Piantadosi CA. Carbon monoxide, reactive oxygen signaling, and oxidative stress. Free Radic Biol Med.2008; 45: 562–569. 10.1016/j.freeradbiomed.2008.05.013 18549826 PMC2570053

[24] Imlay JA. Pathways of oxidative damage. Annu Rev Microbiol. 2003; 57: 395–418. 14527285 10.1146/annurev.micro.57.030502.090938

[25] Thom SR, Marguis RE. Free radical reactions and the inhibitory and lethal actions of high-pressure gases. Undersea Biomed Res. 1987; 14: 485–501. 2825395

[26] Djurhuus R, Svardal AM, Thorsen E. Glutathione in the cellular defense of human lung cells exposed to hyperoxia and high pressure. Undersea Biomed Res. 1999; 26: 75–85. 10372426

[27] Kim YS, Kim SU. "Oligodendroglial cell death induced by oxygen radicals and its protection by catalase". Journal of Neuroscience Research, 29(1):100–6;1991 1886163 10.1002/jnr.490290111

[28] King DW, Smith MA. Proliferative responses observed following vancomycin treatment in renal proximal tubule epithelial cells. Toxicol In Vitro. 2004; 18: 797–803. 15465645 10.1016/j.tiv.2004.03.013

[29] Sokol PP. Mechanism of vancomycin transport in the kidney: studies in rabbit renal brush border and basolateral membrane vesicles. J Pharmacol Exp Ther. 1991; 259: 1283–1287. 1684821

[30] Cisek LH, Gobet RM, Peters CA. Pneumoperitoneum produces reversible renal dysfunction in animals with normal and chronically reduced renal function. J Endourol. 1998; 12: 95–100. 9607433 10.1089/end.1998.12.95

